# Deicer Salt-Scaling Resistance of Concrete Using Recycled Concrete Aggregates Pretreated by Silica Fume Slurry

**DOI:** 10.3390/ma15248874

**Published:** 2022-12-12

**Authors:** Hossein Sasanipour, Farhad Aslani, Javad Taherinezhad

**Affiliations:** 1 Faculty of Civil Engineering, RWTH Aachen University, 52074 Aachen, Germany; 2 Materials and Structures Innovation Group, School of Engineering, University of Western Australia, Perth, WA 6009, Australia; 3 School of Engineering, Edith Cowan University, Perth, WA 6027, Australia; 4 Civil Engineering Department, Bu-Ali Sina University, Hamedan 65178-4161, Iran

**Keywords:** salt-scaling resistance, concrete surface, freeze-thaw cycles, recycled aggregate concrete

## Abstract

**Highlights:**

- Salt-scaling resistance in the presence of recycled concrete aggregates
- A strong bond between RCAs and new mortar was obtained after a freeze-thaw test
- Pulse velocity after exposure to freezing-thawing cycles negligibly decreased
- The scaling rate increased with the increase in the water–cement ratio
- The electrical resistivity of concrete after exposure to cycles has been investigated

**Abstract:**

Concrete wastes such as recycled concrete aggregates (RCA) make up a significant part of construction and demolition waste (C&DW) which can be used to minimize usage of natural aggregates and reduce carbon footprint. This paper studies the salt-scaling resistance of recycled aggregate concrete produced with pretreated RCAs. The test method for evaluating salt-scaling resistance in concrete according to DIN EN 1340: 2003 was performed. Four series of concrete mixes using natural aggregates, RCAs, manually pretreated RCA, and modified RCA in a desiccator were subjected to the different tests in terms of bulk electrical resistance in two directions (X and Y) before and after freeze-thaw cycles, ultrasonic pulse velocity, and weight loss of the surface layer of concrete specimens. Moreover, Scanning Electron Microscopy (SEM) of mixes was conducted and the microstructure of mixes considering the interface transition zone was studied. Results show that after exposure to cycles of freezing and thawing, the quality of concrete regarding ultrasonic pulse velocity did not change. The electrical resistance of specimens decreased significantly in X-direction and slightly in Y-direction after applying freeze-thaw cycles in all mixes. Nevertheless, surface modification of RCAs can increase electrical resistance and improve durability of concrete. SEM images show that the interface transition zone before and after freeze-thaw cycles remained unchanged which means strong bond between aggregate, new mortar, and old mortar. An estimation of the total charge passed indicated that all recycled aggregate concretes can be classified in a safe area and with very low chloride ion penetrability according to ASTM C1202.

## 1. Introduction

In concrete structures, after the mechanical performance, it is necessary to evaluate the durability of concrete. For instance; ion chloride penetration, alkali aggregate reaction, sulfate attack, freeze-thaw damage, salt scaling, to name a few, which are the major concerns for concrete structure [1,2]. Frost resistance durability is an important factor being considered in concrete elements cast in cold regions. Detrimental environmental actions such as freeze-thaw cycles on concrete in cold regions lead to high levels of concrete deterioration [3] including cracking and spalling of concrete due to the progressive expansion of concrete components (paste and mortar) under Abrupt temperature changes [2,4,5]. Due to frost and manifestation of internal microcracking, the mechanical properties of concrete are lost, leading to destruction of structure. In the presence of water on the surface of concrete in cold regions, surface scaling can occur attributed mainly to hydraulic pressure of water in the pore system during crystallization. Additionally, salt scaling as a major durability issue is a superficial and progressive damage in concrete infrastructures causing severe scaling or flaking of the surface by lowering the melting point of saline solution which magnifies the susceptibility of concrete [6,7,8]. The most part of concrete’s surface consists of more paste rather than the body of concrete. Ordinary in the surface of concrete, the water–cement ration is higher than that of other parts because of bleeding. In addition, micro-cracks induced by shrinkage can be found on the surface. Weak paste and micro-cracks lead to low salt-scaling resistance of concrete [5]. This situation could be more severe, whereas the recycled aggregates are associated as partial replacement of fine or coarse natural aggregates due mainly to the inferior physical and mechanical properties of RCAs. Construction activities consume a huge amount of aggregates derived from natural resources accounting for a significant portion of global CO_2_ emissions. Marvila et al. [9], reported that 4.5 bi tons of solid waste including C&DW, ceramic, glass, slag, and polyethylene terephthalate (PET), are being generated every year. The important proportion of waste materials accounting for 35% is disposed of in landfill. Incorporating RCAs in concrete production is an advantageous and promising solution which can mitigate these environmental problems [9].

It is expected that more levels of damage consisting of more cracks, greater mass loss, surface scaling, and spalling occur whereas RCAs are incorporated in concrete mixes culminating in further penetration of aggressive agents (chloride, sulfate, etc.) [10,11]. Although there are limited applications of RCAs owing to the higher porosity and lower mechanical characteristics, the results in the literature show that RCAs can be used in structural concrete [3,12,13,14]. However, for recycled aggregate concrete to have satisfying performance in cold regions and acceptable frost resistance, some provisions can be considered. Reports concur that the amount of adhered mortar, water–cement ratio of original concrete, and replacement ratio of RCAs can adversely affect freeze-thaw resistance. At the opposite pole, using RCAs—extracted from high strength original concrete, pre-soaking methods, and treatment methods of RCA—additives such as mineral and chemical materials can favorably improve freeze-thaw resistance [10,11,15,16].

## 2. Significance of the Research

Although the concise state-of-the-art review presented in the literature indicated that there have been a wide range of studies related to recycled aggregate concrete (RAC) performance, whether the treated RCAs can affect the durability performance of concrete in extreme climate events and hostile environments is unknown for sure. As only a limited number of technical papers related to the topic of the current manuscript can be found in the literature, the investigation of recycled aggregate concrete’s performance subjected to freeze-thaw cycles in the presence of deicing salts seems to be worthwhile (in cold regions, salts are regularly used to deice concrete walkways and roadways). In addition, due to the significant heterogeneity of recycled concrete aggregates derived from different origin concrete, the amount of attached mortar, and other negative characteristics of RCA, durability aspects of concretes made with recycled concrete aggregates (RCAs) must be considered before any practical applications [17].

Using treatment methods for enhancing properties of RCAs is an advantage to mitigate the challenges of concrete mixes casting with RCAs. There are found to be several methods for improving by many scholars conducted to date, the most important of which are (1) strengthening of attached mortar (AM) and (2) removing of AM [18]. A review on different treatment methods indicated that strengthening of AM is also a cost-effective, eco-friendly and sustainable method in comparison with the latter [19]. A reinforced AM can be reached by introducing nano-materials and pozzolans in concrete mixes culminating in an environmentally friendly approach for improving the properties of recycled aggregates [19].

Specifically, this paper deals with the salt-scaling resistance of high-strength concretes consisting of conventional concrete (C60-C75) prepared with natural aggregates and recycled aggregate concretes (C50-C60) produced with RCAs which are untreated and treated by silica fume slurry around RCA’s surfaces. To this end, two surface modification methods to treat the surface of RCAs employed herein were considered with the aim to investigate the effect of the modification method on the salt-scaling resistance of mixes.

## 3. Materials, Modification and Test Methods

**Figure 1:**
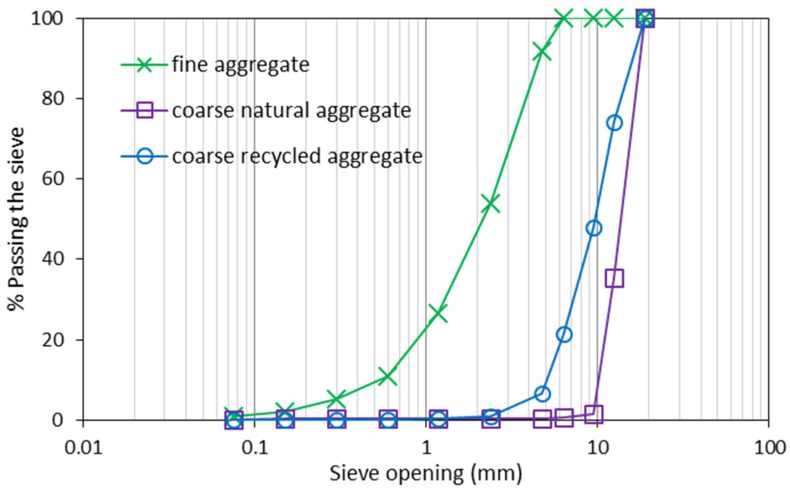
Gradation of the fine and coarse aggregates.

Portland cement type Ⅱ and silica fume (SF) as supplementary cementitious materials and also for coating the surface of RCAs were used in this study. Properties of cement and silica fume are given in Table 1 and Table 2. Preparation of recycled concrete aggregates was conducted in the laboratory using crushing machine. Based on ASTM C127 [20], the specific gravity and water absorption of coarse RCAs were recorded at 2.39 and 5.4%, respectively. The specific gravity of 2.63 for both fine and coarse natural aggregates and water absorption of 1.7% and 2.61%, respectively, were measured. Limestone powder was used as filling material with the specific gravity of 2.58 because the fine aggregate used in mixes was not fine enough. Properties of aggregates used in the study and particle distribution of aggregates is shown in Table 3 and Figure 1, respectively. To measure adhered mortar content, the thermal method developed by Marta Sánchez and Pilar Alaejos [21], was employed in this study.

To provide convenient workability, a chemical admixture based on ether polycarboxylate was used and the quantity of superplasticizer was kept (1.1% by weight of cement in all mixes) which helps to eliminate the effect of chemical admixture on the durability characteristic of mixes.

Recycled concrete aggregates were modified to strengthen adhered mortar on the aggregates. Two modification methods were considered in which silica fume slurry including 250 gr SF and 1000 cc water added to 8 kg of RCA. In the first method called RACCM, RCAs are coated by SF slurry manually. The RCAs were poured into SF slurry and the coated aggregates were transferred to the oven and dried for 72 h at 45 °C. For the second modification method called RACCD (Coated in Desiccator) to be used in mixes, RCAs were poured in a desiccator in a dry manner for 2 h and then SF slurry was added to the desiccator to seal the surface of RCAs in the desiccator for one hour. After which, RCAs were transferred to an oven-dried machine, the same as the RACCM method.

**Figure 2:**
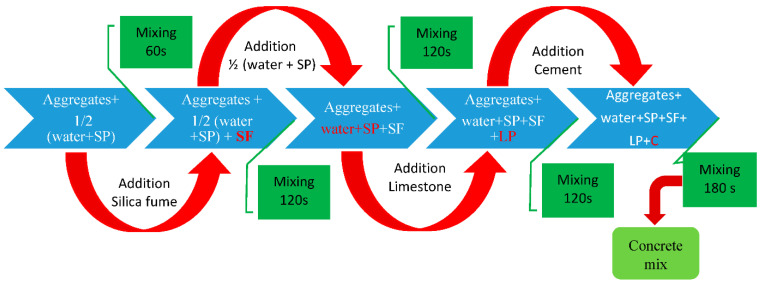
Mix procedure of concrete mixes.

This study aims to investigate the effect of deicing salts in freeze-thaw cycles on four series of mixes including normal concrete, recycled concrete with untreated aggregates, and two series of recycled concretes using pretreated RCAs in which the absolute volume method for concrete mix design was employed. Coarse RCAs with replacement of 100% were prepared in a laboratory using a small rock crushing machine. To separate impurities from the surface of RCAs, they were presoaked in water before mixing in concretes containing untreated recycled concrete aggregates. Table 4 shows the mix proportion in the saturated surface dry (SSD) situation and compressive strength of mixes at 28 days. In addition, the stages of concrete mix procedure are presented in Figure 2.

**Figure 3:**
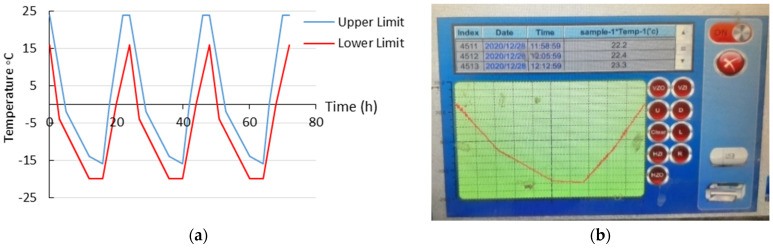
(**a**) Upper and lower limits of cycles of freezing and thawing and (**b**) applied cycles in experimental study.

Freeze-thaw tests according to DIN EN 1340: 2003 [22] were conducted on three specimens in each series. Figure 3a shows the upper and lower limit of temperature in 24 h as a completed cycle of freezing and thawing, and Figure 3b indicates the real temperature of one cycle of freeze-thaw test set up in the laboratory. To investigate the resistance of concrete in present of deicer salts (3% NaCl), freeze-thaw tests were performed on cube 100 × 100 × 50 (height) mm specimens cut from 100 × 100 × 100 mm cubes. The specimens were covered with moisture isolation and thermal insulation to prevent the effect of freeze-thaw cycles on the other surfaces. Moisture isolation were selected from flexible materials somehow during freeze-thaw cycles extra tension did not apply to specimens. Mass loss of samples after exposure to cycles of freezing and thawing was measured in every five cycles to draw the scaling profile.

**Figure 4:**
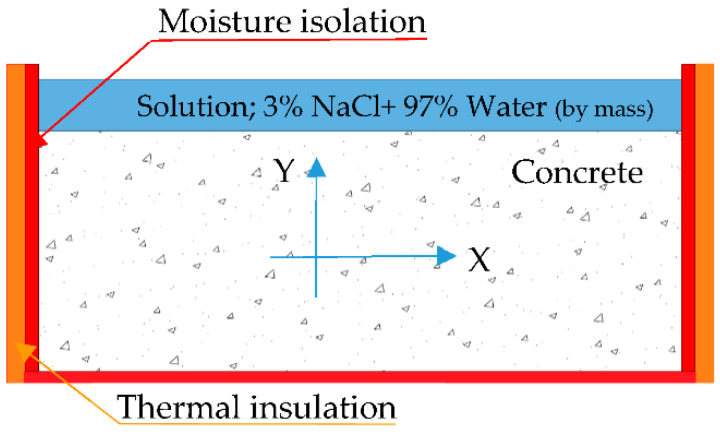
Schematic section of freeze-thaw specimen, electrical resistance measurement’s direction, and amount of solutions on the surface.

**Figure 5:**
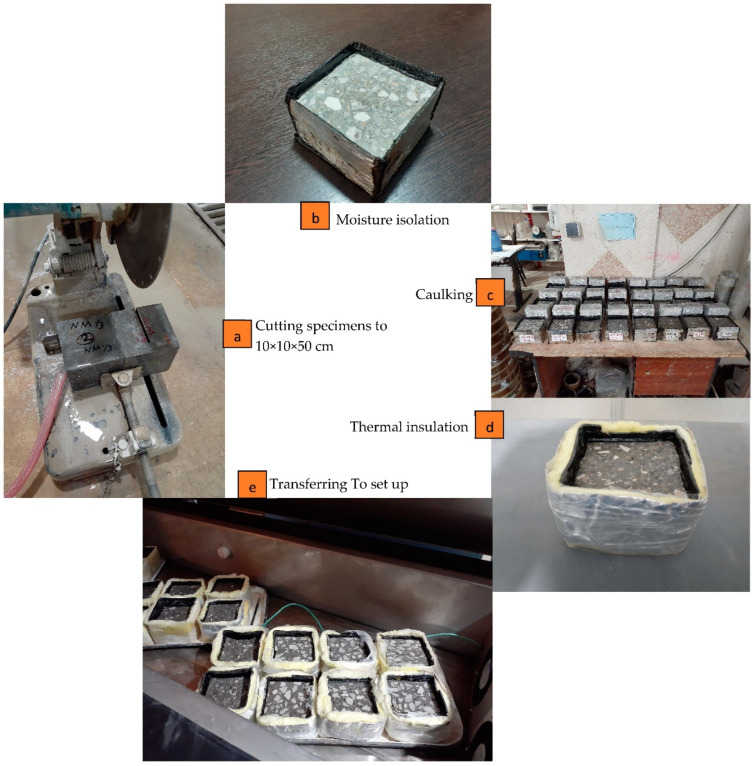
Preparation stages of freeze-thaw test samples.

**Figure 6:**
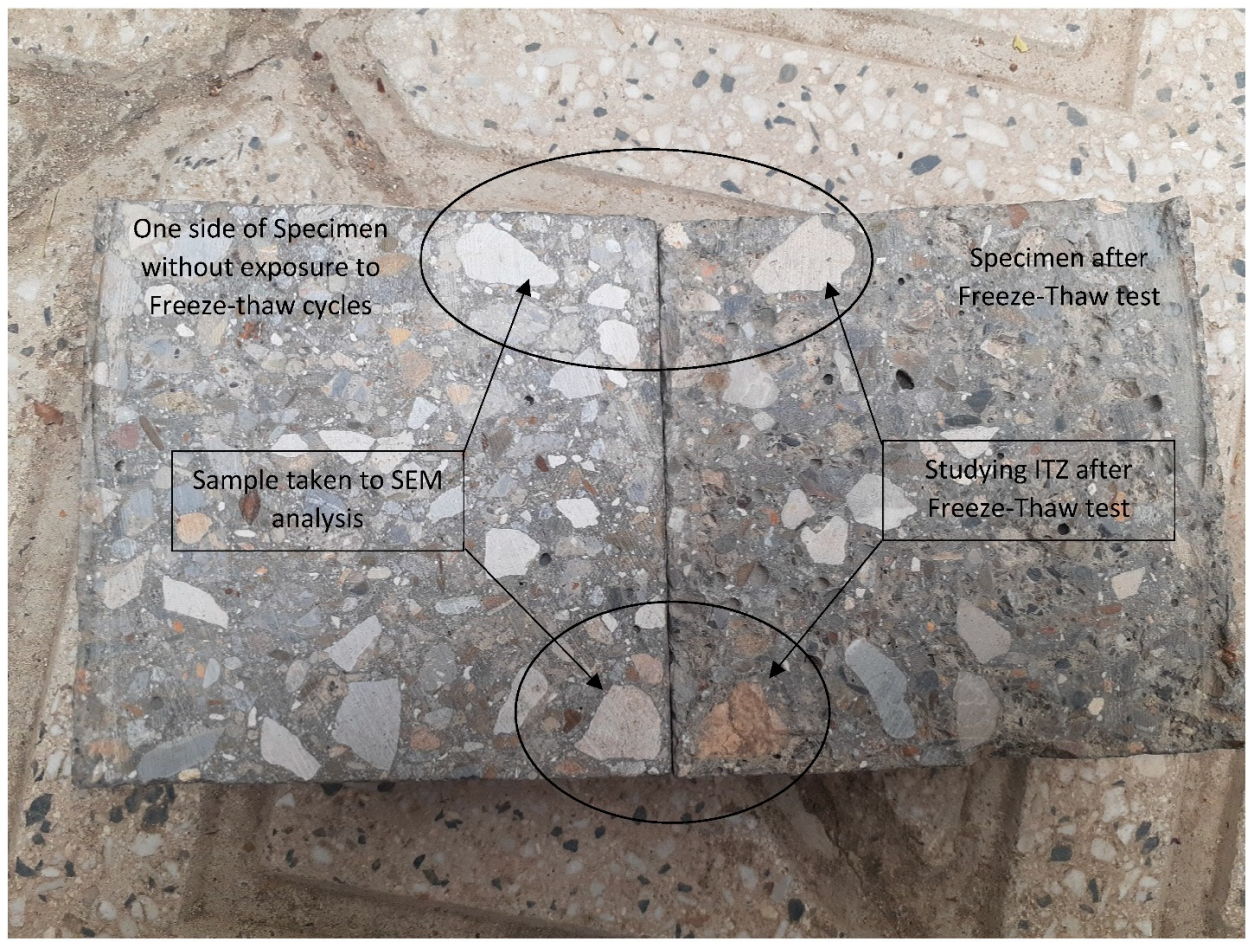
Studying the microstructure of samples taken from a common place to evaluate the effect of deicer salts and freeze-thaw cycles.

To measure the pulse velocity of samples before and after the freeze-thaw test, a portable ultrasonic non-destructive digital indicating tester (PUNDIT) was used. The bulk electrical resistance of mixes was conducted on specimens used in the freeze-thaw test before and after applying 28 cycles. The blue vectors in Figure 4 show the direction of the ultrasound in the ultrasonic pulse velocity path and the ions movement in electrical resistance measurement. Figure 5 shows the preparation procedure of samples from cutting to placing samples in set up. In addition, SEM analysis were prepared on samples for each mix taken from a common place of specimen (See Figure 6). Then, samples were carefully cut, polished, dried and coated with gold to take SEM images.

## 4. Results and Discussion

### 4.1. Visual Laboratory Observations

**Figure 7:**
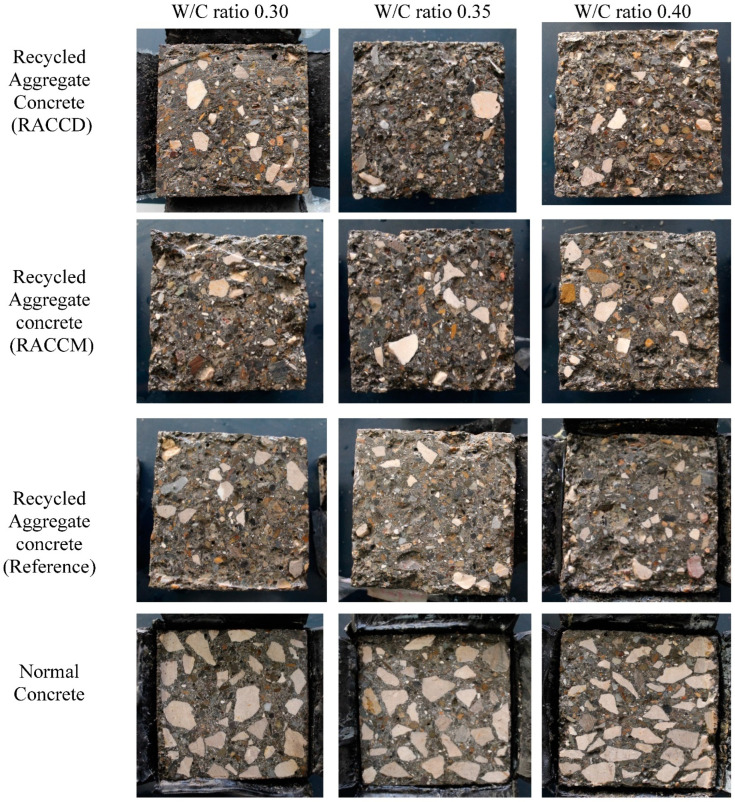
Surface appearances of RAC Treatment method (RACCD), RAC Treatment method (RACCM), RAC (Reference), Normal Concrete specimens after exposure to 28 cycles of freezing and thawing in the presence of deicer salt.

**Figure 8:**
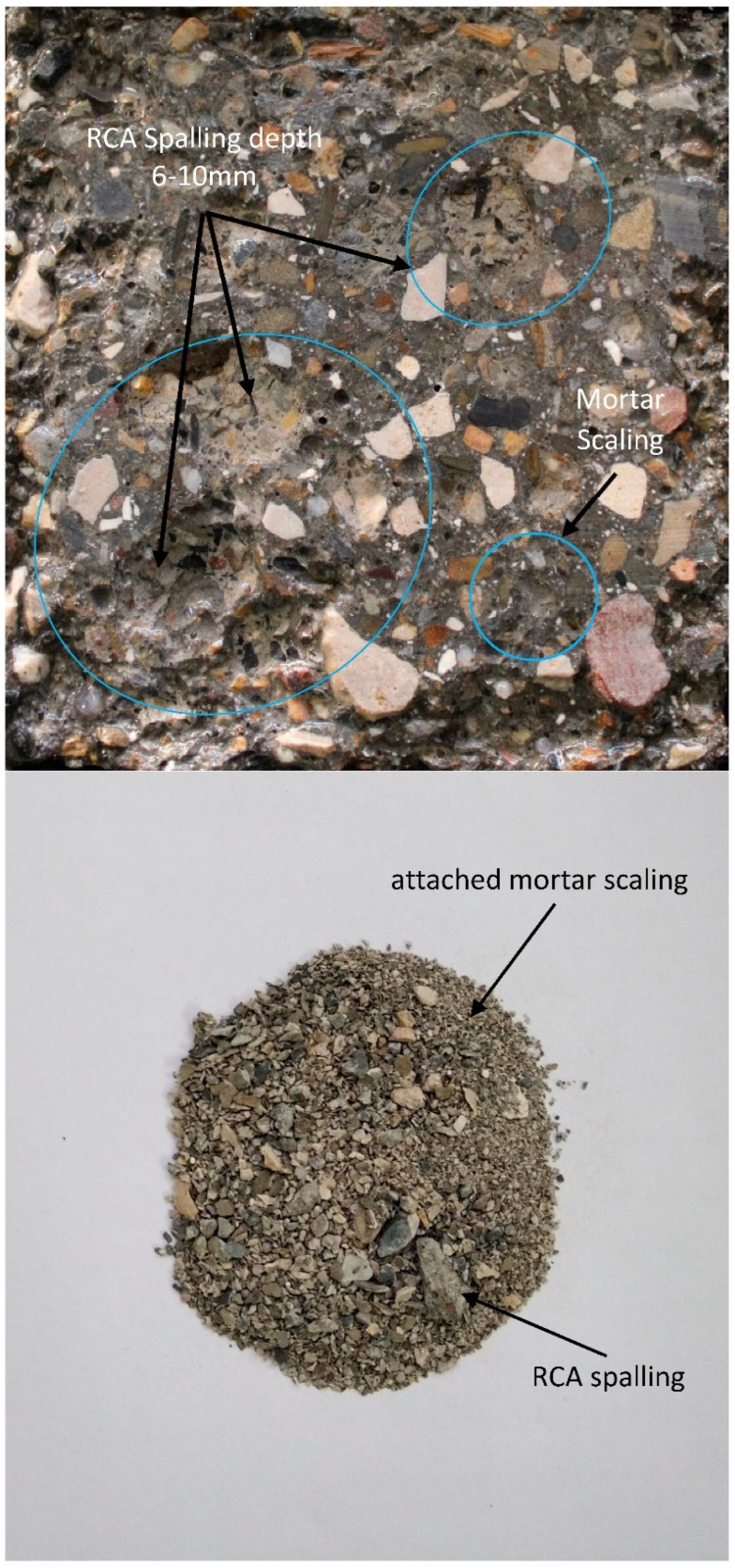
RCA spalling and mortar scaling of mix containing recycled concrete aggregate.

Figure 7 shows the concrete’s surface after applying 28 cycles of freezing and thawing. After 28 repeated cycles, concretes with natural aggregates exhibited minor salt scaling. Incorporating RCAs in mixes containing recycled aggregates leads to an increase in surface damage. Having applied the freeze-thaw cycles, a severe surface scaling was observed in the old mortar and recycled aggregates; whereas the mass loss of paste and RCAs in a depth of approximately 6–11 mm (Figure 8) was seen. “In certain cases where scaling is very severe, the loss of coarse aggregate particles is possible and the concrete can be affected to a depth of more than 1–2 cm [5]”. Figure 7 also compares the effect of the water–cement ratio. With the increase in the water–cement ratio, the surface damage increased, which can be attributed to the bleeding, weak paste, and more porous structure.

### 4.2. Mass Loss

**Figure 9:**
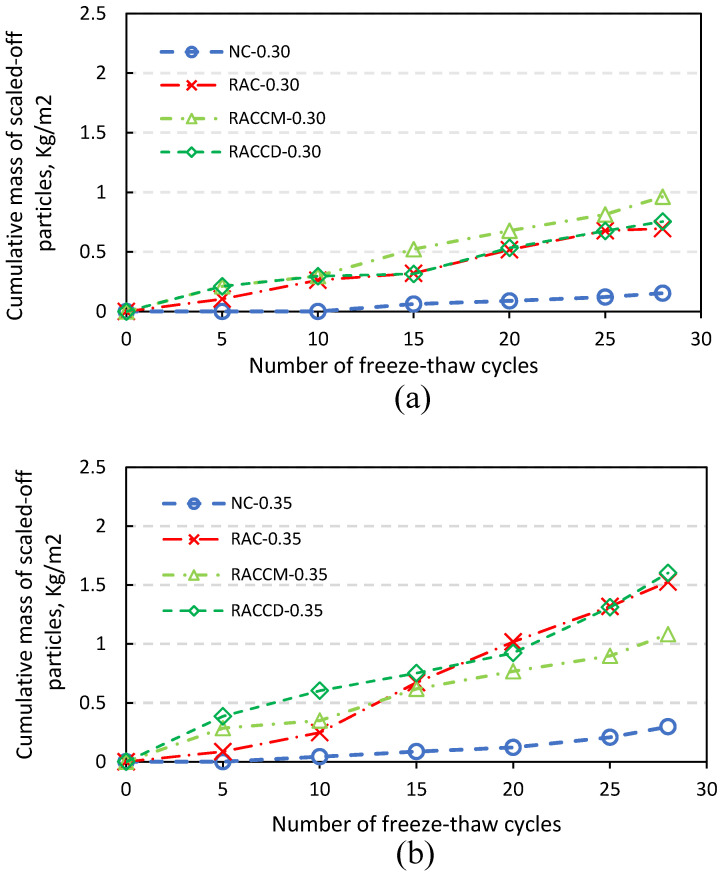

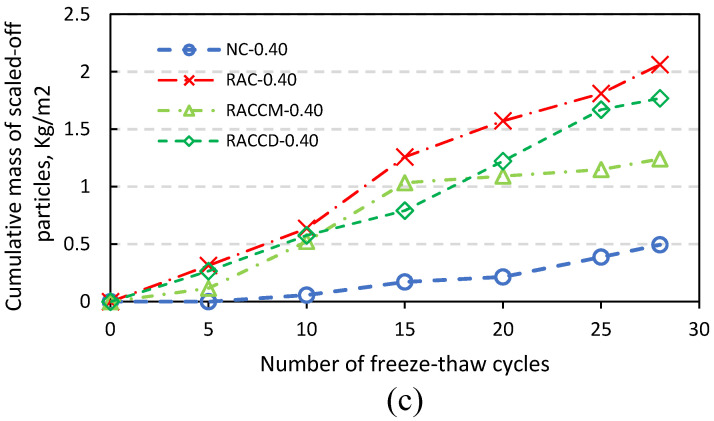
Cumulative mass of scaled-off residues of mixes: (**a**) w/c 0.30, (**b**) w/c 0.35, and (**c**) w/c 0.40.

The cumulative mass of scaled-off particles (kg/m^2^) from the surface of specimens after exposure to freeze-thaw cycles in the present of deicer salt can be found in Figure 9. It is crystal clear that the major scaling belongs to mixes containing recycled aggregates. The pore structure of mortar adhered to RCAs makes it easy for a large amount of water to penetrate in voids resulting in more signs of the specimen’s damage during cycles. Recycled aggregate concrete is generally characterized by a higher porosity than conventional concrete so that freeze-thaw resistance and mass loss are lower and greater, respectively, than mixes casting with natural aggregates [23]. In addition, reviewing the literature shows that the number and the volume of capillary pores can be the main cause of the expansive internal pressure during freezing resulting in more mass loss [24,25]. Physical deterioration mechanisms during cycles of freezing and thawing can be explained as follows [5,26]; with the decrease of temperature, water in the capillary pores of concrete goes gradually to be formed as the ice. Whereas the temperature is under 0 °C, the water freezing process will be beginning in the capillaries, thereby increasing the volume of water (with a 9% increase of volume in the transition from liquid to solid). In high saturation degree of cement paste, the water is penetrating in the capillary pores until they are full. As the pores cannot be expanded, a certain amount of water is forced out. To abate the pressure caused by water, it should move into available places; in this case, a large number of air voids (connected to capillary pores which are full) is needed to move the water from capillaries. Therefore, water can be frozen in air voids without causing any damage. Otherwise, the capillary pores are full and because of the freezing rate, a certain pressure will be applied around the internal layer of voids. If the tensile strength of paste is less than the internal stress applied by ice, the paste breaks in tension. The connectivity between pores is the most important factor which can improve the freeze-thaw resistance. It is proven that the higher the connectivity of the macropores, the higher the freeze-thaw resistance of the mortar [27]. In this regard, incorporating air entrained agents improves the freeze-thaw resistance [7]. The MgO expansion agent, fly ash, and PVA fiber can also be used to enhance the impermeability of concrete resulting in more densified pore structure and low cracking risk of concrete under abrupt temperature changes [28,29,30].

In the presence of deicing salts, the amount of ice formed in pores could be reduced and the literature review shows the salt solution concentrated on the concrete surface is more important than the composition of the pore liquid [31,32]. Added to this, it is reported that damages caused by deicing salts are often progressive removal of small flakes or chips of binder without a significant loss of strength of concrete [33,34].

As illustrated in Figure 9a, the mass of normal concrete remained approximately unchanged under freeze-thaw cycles which means high salt-scaling resistance; whereas, concretes containing RCAs represent 4.5 (RAC), 6.2 (RACCM), and 4.9 (RACCD) times mass loss as much as that in normal concrete at the end of test. Moreover, concretes with w/c ratios 0.35 and 0.40 (Figure 9b,c) experienced more damage on their surface in comparison with mixes in a w/c ratio of 0.30. It can be attributed to the increase in permeable voids whereas the w/c ratio is increased resulting in more tension with the increase in the freezing rate which means more physical deterioration. In case of the w/c ratio of 0.35, the number of RCAs on the surface of specimen RACCD was unexpectedly higher than that in others (RACCM and RAC) and consequently, the cumulative mass of scaled-off particles exceeds than that of other specimens. The highest rank of deterioration goes to a w/c ratio 0.40 with the major damage accounting for the mix containing un-pretreated recycled aggregates. Minsoo Kim et al. [35] reported that due to the increase in the effective w/c ratio, weaker cementitious matrix can be formed, and consequently internal damage and salt scaling could be increased. They also found that with the increase in the effective w/c ratio, the amount of water can penetrate in a unit volume of mortar causing more damage.

### 4.3. Ultrasonic Pulse Velocity

**Figure 10:**
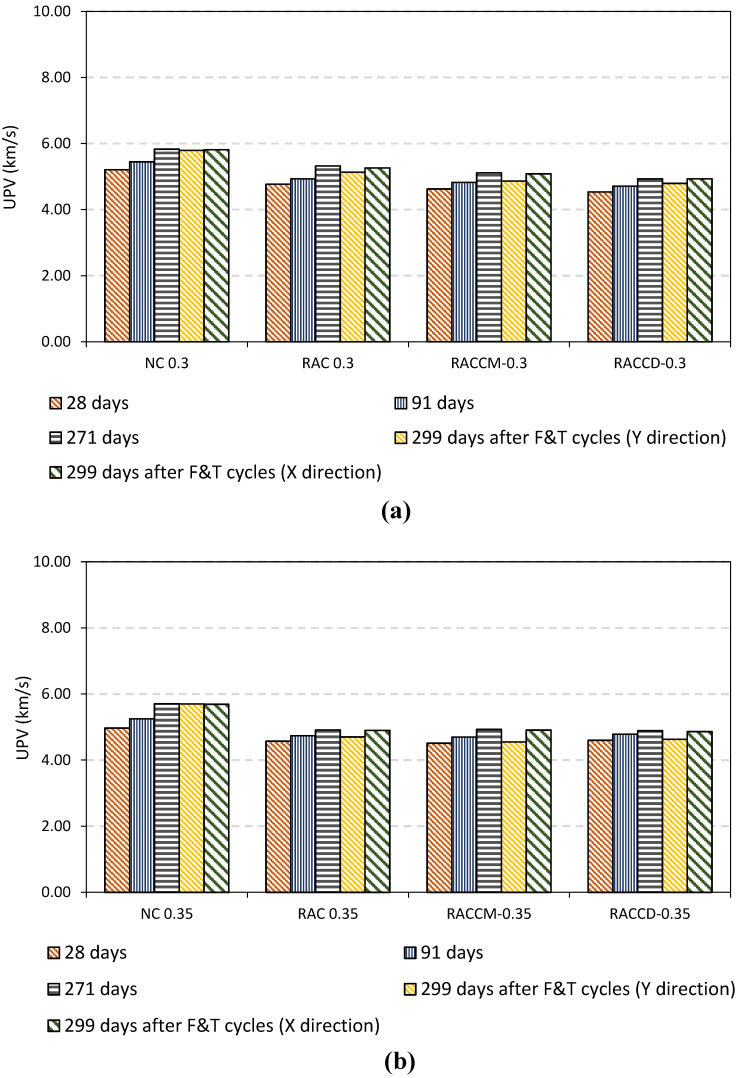

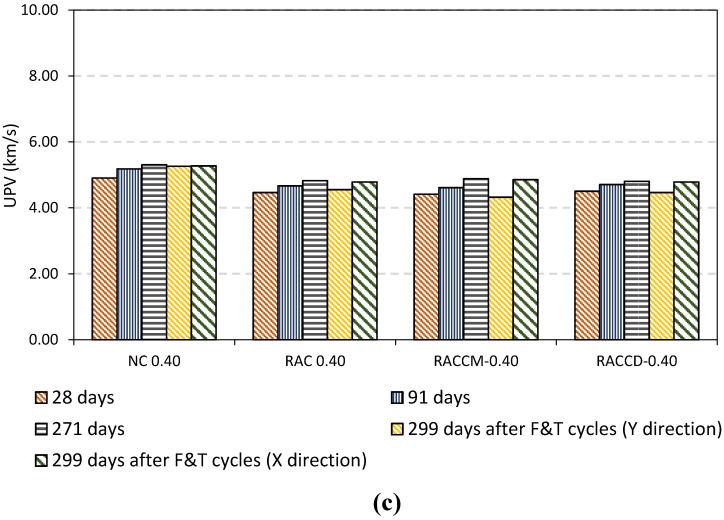
The ultrasonic pulse velocity results before and after exposure to freeze-thaw; (**a**) w/c ratio 0.30, (**b**) w/c ratio 0.35, and (**c**) w/c ratio 0.40.

Figure 10 shows the UPV of mixes before (28, 91, and 271 days) and after exposure to cycles of freezing-thawing in X- and Y-directions. Incorporating RCAs in all mixes reduced UPV. In addition, it can be found that an increase in the w/c ratio results in lower UPV of mixes (See Figure 10b,c). Reviewing the literature shows that the porosity can affect UPV, so that a higher w/c and a large amount of RCAs used in mixes culminate in reduction of pulse velocity [36,37].

As illustrated in Figure 10, the UPV of concretes after exposure to freeze-thaw cycles experienced a negligible reduction between 0.30–1.30 percent in X-direction. Previous studies reveal that UPV can be changed to associate with interface transition zone quality and concrete density [23,38,39]. As shown in Figure 10, the UPV of concretes decreased slowly after applying cycles. It can be concluded that cycles of freezing and thawing have no extreme impact on the physical properties, emerging, or developing cracks in the depth of concrete specimens; whereas the UPV of specimens was measured in X-direction. UPV measured in Y-direction shows that a greater reduction of pulse velocity can be found in mixes containing recycled aggregates which can be attributed to the spalled and scaled surface of RAC mixes (See Figure 8). Due to spalling or scaling and microcracks on the surface, resulting from cycles of freezing and thawing, UPV decreased between 4–6% in the RAC group, 5–11% in the RACCM group, and 3–7% in the RACCD group. Those reductions are directly related to the probability of distribution of recycled concrete aggregates on the surface of specimens resulting in an unforeseen decrease in each group. The results of the UPV test and a comparison between results before and after applying cycles show that the damage is related to the surface of specimens and deep cracks cannot occur in the path of ultrasound pulse measurement. The surface scaling in specimens leads to a poor contact between concrete and transducers resulting in a reduction in UPV and lower freeze-thaw resistance even if no significant internal microcracking occurs [5].

Previous studies of authors show that the quality of all mixes in terms of pulse velocity at the age of 91, according to BIS 13311–92, were classified in the excellent categories of concrete [40,41]. However, after exposure to freeze-thaw cycles, the values of UPV are around 4.5 km/s which remained in excellent level of classification.

### 4.4. Microstructure Study

**Figure 11:**
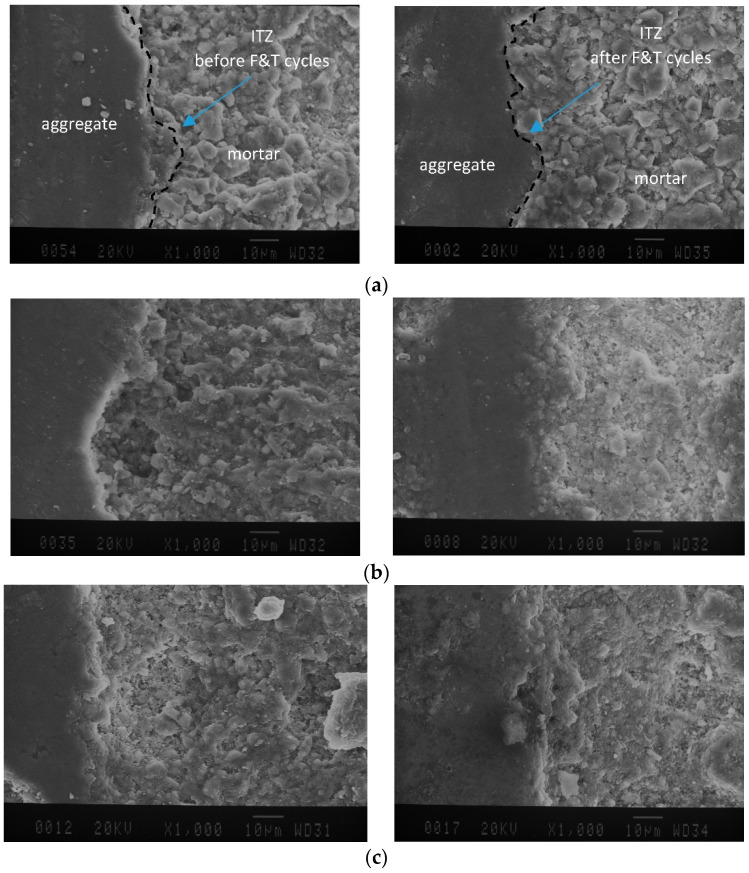
SEM images of ITZ before (**left**) and after (**right**) applying freeze-thaw cycles; (**a**) mix including pretreated RCA in desiccator, (**b**) mix using manually pretreated RCA, and (**c**) mix containing untreated RCA.; (**a**) RACCD w/c ratio 0.30, (**b**) RACCM w/c ratio 0.30, and (**c**) RAC w/c ratio 0.30.

Figure 11 depicts SEM images of specimens with a w/c ration of 0.30 before and after applying 28 cycles of freezing and thawing in the present of deicer salt. As illustrated in Figure 11, the quality of ITZ in specimens has comparatively not been changed after exposure to cycles. In addition, SEM images of other groups, including a w/c ratio of 0.35 and 0.40, indicated a strong bond between aggregates after specimen exposure to freeze-thaw cycles. These trends seem to be supported by the formation of new hydrated compounds (C-S-H) resulted from pozzolanic activity of supplementary cementitious materials such as silica fume and slag in early or late period of hydration [7]. New C-S-H produced through cement hydration acts to fill pores and voids resulting in strengthening the interface transition zone, and leading to a decrease in the size of internal pores which makes a strong bond between paste-aggregate and old mortar-new mortar [42,43,44]. Moreover, the compactness of the ITZs can be achieved by filling effects of new hydrated compounds culminating in superior mechanical performance and durability [45].

**Figure 12:**
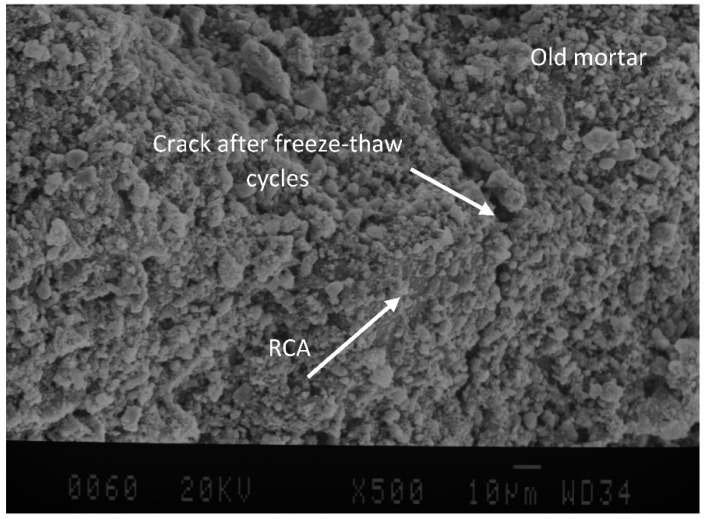
Surface deterioration of recycled concrete aggregates exposure to freeze and thaw cycles.

Figure 11 clearly indicates that after exposure to cycles, a denser microstructure with a low number of pores near the ITZ can be obtained. However, due to the freezing-thawing rate during cycles, on the surface of old mortar in RCAs some cracks can be seen. Existing micro-cracks, inferior durability, and high level of porosity in adhered mortar [11] makes essay surface deterioration of recycled concrete aggregates develop (See Figure 12).

**Figure 13:**
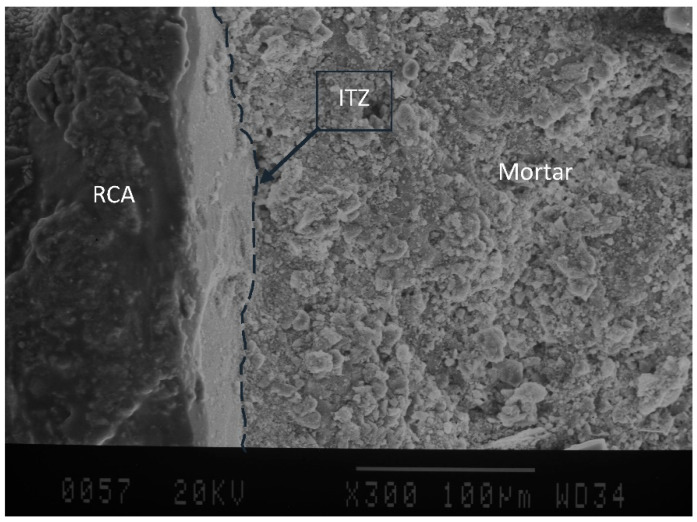
Spalled RCA and strong bond between RCA and new mortar.

Visual observation in the laboratory showed that spalling of RCAs started from the upper layer of cut RCAs on the surface of specimens and continued to the vicinity of new past (See Figure 13); whereas a strong bound between RCA and new mortar was obtained. SEM images from a previous study of authors [40] indicated that at 91 days, using SF and pretreated RCAs in mixes leads to consumption of accumulated Ca(OH)_2_ near ITZ and formation of new hydration products that improve the interface in the microstructure.

### 4.5. Electrical Resistance

**Figure 14:**
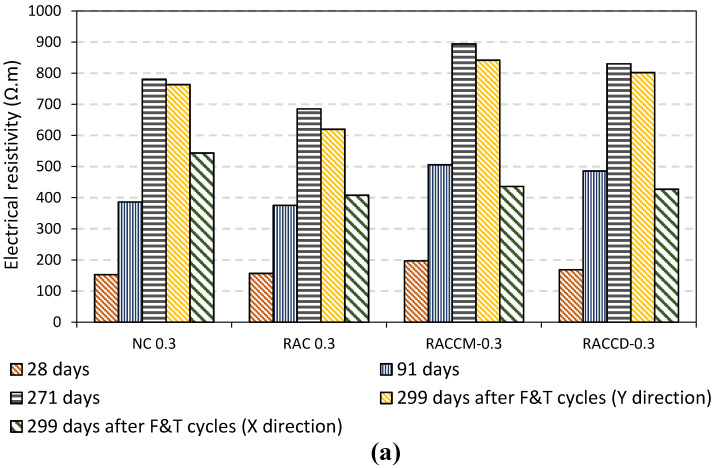

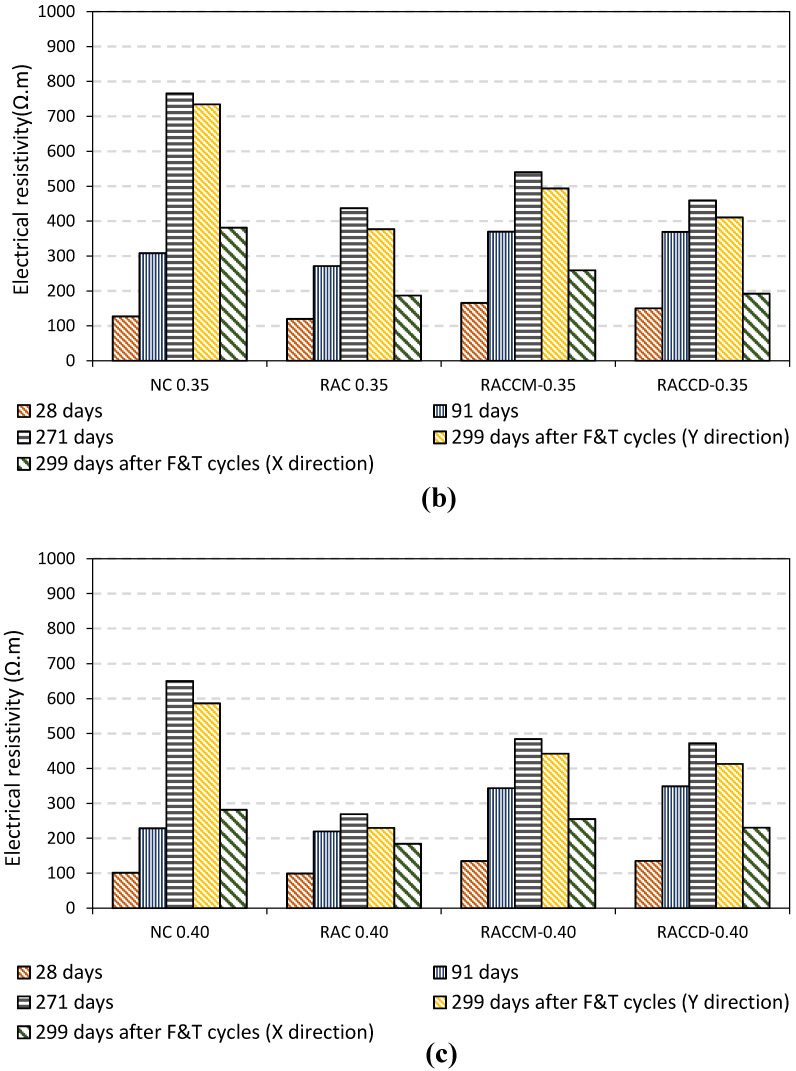
Electrical resistivity of mixes before and after exposure to cycles of freezing and thawing; (**a**) w-c ration 0.30, (**b**) w-c ration 0.35, (**c**) w-c ration 0.40.

The electrical resistivity (ER) values of concretes at 28, 91, 271 (before applying cycles), and 299 days (after exposure to cycles of freezing and thawing in X- and Y-direction) are shown in Figure 14. As shown in the figures, the ER of all mixes increased with the increase in the age which can be related to the completion of hydration in cement, silica fume, and other cement components. However, after exposure to cycles, a sharp reduction occurred in X-direction which seems to be unconscionable. Reviewing the results of UPV tests and SEM images in this study indicate that the reduction in electrical resistivity is not related to a physical mechanism. Although accessible papers regarding the effect of freeze-thaw cycles on electrical resistance are very limited in the literature, generally, the electrical resistance decreases with frost damages of concrete under abrupt temperature changes [46]. It is expected that the reduction of electrical resistivity in X-direction mostly depends on NaCl solution penetrated in pores of mortar and aggregates. A decrease in electrical resistivity can be obtained whereas the concentration of NaCl increases on the surface. Due to the ionic concentration, the electrical conductivity increases, thereby decreasing the electrical resistance of specimens [47]. The increase in the degree of saturation and NaCl deicing salt concentration causes a sharp reduction of electrical resistance in concrete specimens. In fact, as the amounts of chloride ions, NaCl salt, or sodium in the pore solution increase, the electrical resistivity of pore solution decreases.

Turning to the details of bulk electrical resistance, results in Y-direction depict that all mixes resist against ion movement. However, a slight reduction of ER can be seen in mixes ranging 2–10% in normal concrete, 9.5–15% in the RAC group, 6–9% in RACCM, and 3–13% in the RACCD group. Decreasing in degree of saturation of the matrix and also other components of concrete such as aggregates, the electrical resistivity of the complex increases; “this is due to the reduction in resistivity of the pore solution (the conductive medium) and increase in tortuosity” [47].

**Figure 15:**
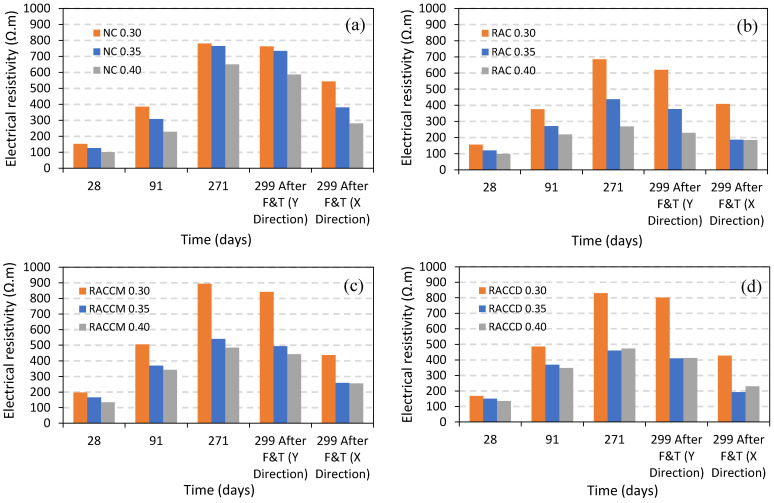
Effect of water–cement ratio on electrical resistivity of mixes before and after exposure to cycles of freezing and thawing; (**a**) normal concrete, (**b**) RAC group, (**c**) RACCM group, and (**d**) RACCD group.

Figure 15 also illustrates the effect of the water–cement ratio on the electrical resistance of mixes. As it can be seen, an increase in w/c ratio decreases ER in most of the mixes which can be attributed to the more porous structure of the matrix, the weak interface transition zone between paste, and aggregates. It is worthwhile to mention that in all mixes, the value of ER in Y-direction after exposure to cycles of freezing and thawing is more than 400 Ω.m which means a durable concrete in terms of corrosion resistance induced by chlorides. It is widely accepted that the corrosion rate can be evaluated by electrical resistivity measurement of concrete which is easily and reasonably priced [48]. According to the research by Cavalier and Vassie [49], a corrosion rate using half-cell tests were usually not significant; whereas, concrete resistivity is bigger than 12,000 ohm-cm. In another technical paper published in 1993 by López and Gonzále [50], concrete with electrical resistivity between 300–400 Ω.m had a low rate of corrosion intensity. Morris et al. also indicated that a passive state can be reached for steel reinforcement when the electrical resistivity is higher than 30 kΩ.cm [51].

### 4.6. Estimation of Total Charge Passed

**Figure 16:**
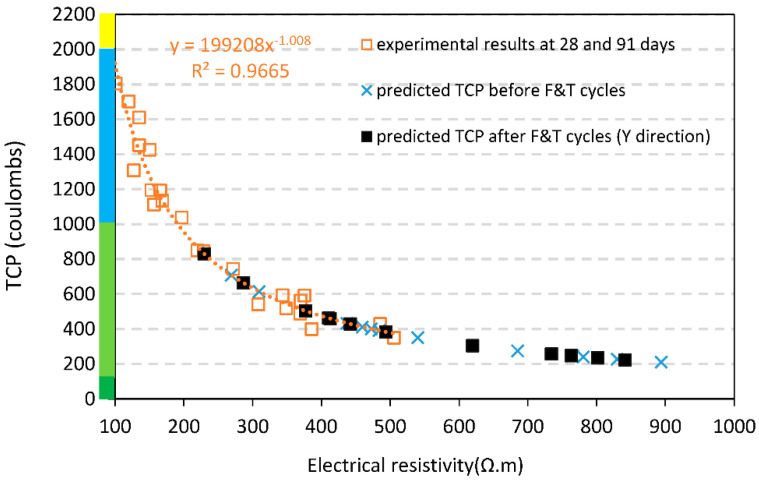
Estimation of total charge passed based on experimental results and equation obtained from last technical paper [40].

According to the last investigation [40], authors obtained a strong correlation between a total charge passed (TCP) based rapid chloride penetration test [52] and bulk electrical resistivity with a wide range of data including 28 and 91 experimental results. The power function ($y=199208\;x^{-1.008}\;with\;R^{2}=0.97)$ indicated that the greater values of ER represent the greater resistance of concrete against the ions’ movements in terms of TCP. Regarding the equation and electrical resistivity derived from specimen’s tests before and after exposure to cycles of freezing-thawing, the values of TCP can be predicted (See Figure 16). As illustrated in Figure 16, after applying freeze-thaw cycles, chloride ion penetrability (CPI) remains in a safe area ranging between 200 and 850 coulombs allocated to the *Very Low* CIP classification of ASTM C1202 [52].

## 5. Conclusions

In this paper deicer salt-scaling resistance of recycled aggregate concrete was evaluated. Concrete mixtures were prepared with natural aggregates, untreated RCAs, and pretreated RCAs. To strengthen adhered mortar on the surface of RCAs, silica fume slurry was used. The following results were derived.

The data presented herein demonstrated that in the presence of deicing salts, recycled aggregate concrete is not resistant to scaling. However, it would be possible to utilize pretreated RCAs in applications exposed to freeze-thaw cycles providing a protective layer of conventional concrete on top of which recycled aggregate concrete is cast.

After exposure to cycles of freezing and thawing, electrical resistivity of mixes decreased sharply in X-direction. The main reason for electrical resistivity reduction is ionic concentration on the surface of specimens in the presence of NaCl which facilitates ion movements. Electrical resistivity in Y-direction decreased after applying cycles in mixes containing RCAs which can be associates with frost damages of concrete under abrupt temperature changes. However, surface modification of recycled aggregate can assist in improving the electrical resistivity which means a beneficial effect on durability in terms of corrosion. Estimation of total charge passed of mixes after exposure to cycles indicated that pretreated recycled aggregates can resist against ion movements. The chloride ion penetrability remains in a safe area according to ASTM C1202. The ultrasound pulse velocity of recycled aggregate concrete after exposure to cycles of freezing and thawing can be classified in the excellent category in terms of ultrasonic response. The presence of RCAs did not follow a negative effect on the interface transition zone of specimens after exposure to cycles which means RCAs exhibited a strong bond with new mortar. However, some cracks on the surface of adhered mortar resulting from abrupt temperature changes can be found.

## Figures and Tables

**Table 1:** Chemical properties of cement and silica fume.

Chemical Properties	Cement	Silica Fume
	(%)	(%)
SiO_2_	21.27	85–95
CaO	62.95	-
Fe_2_O_3_	4.03	0.4–2
Al_2_O_3_	4.95	0.5–1.7
MgO	1.55	0.1–0.9
Na_2_O	0.49	0.15–0.2
K_2_O	0.65	0.15–1.02
SO_3_	2.26	-
C_3_A	6.3	-
LOI	2.11	3.5

**Table 2:** Physical and mechanical properties of cement and silica fume.

Physical Properties	Cement	Silica Fume
Specific gravity	3–3.1	2.21
Specific surface (cm^2^/gr)	2910	14,000
Setting Time (min)	Initial—154	-
	Final—195	-
**Mortar Compressive strength (MPa)**		
*f’c* 3 days	20.1	-
*f’c* 7 days	28.2	-
*f’c* 28 days	40.3	-

**Table 3:** Properties of RCA and natural aggregates.

Type of Aggregate		Density (g/cm^3^)	Maximum Size (mm)	Fineness Modulus	Water Absorption (%)	Los Angeles Abrasion (%)	Adhered Mortar (%)
Coarse	Natural	2.63	19	-	1.7	15.2	-
	RCA	2.39	19	-	5.4	33.1	37.8
Fine	Natural	2.63	-	3.88	2.61	-	-

**Table 4:** Mix proportion, slump, and compressive strength.

Mix Code	W/C	C	W	SF	Coarse Natural Aggregate	Coarse Recycled Aggregate	Fine Aggregate	Limestone Powder	SP (%)	Compressive Strength (MPa)
		Kg/m^3^								
NC 0.30	0.30	386.4	126	33.6	750	-	938	184	1.1	78.1
NC 0.35	0.35	386.4	147	33.6	728	-	911	179	1.1	72.6
NC 0.40	0.40	386.4	168	33.6	706	-	883	173	1.1	58.9
RAC 0.30	0.30	386.4	126	33.6	-	682	938	184	1.1	60.3
RAC 0.35	0.35	386.4	147	33.6	-	662	911	179	1.1	57.2
RAC 0.40	0.40	386.4	168	33.6	-	641	883	173	1.1	55.5
RACCM 0.30	0.30	386.4	126	33.6	-	682	938	184	1.1	61
RACCM 0.35	0.35	386.4	147	33.6	-	662	911	179	1.1	54.5
RACCM 0.40	0.40	386.4	168	33.6	-	641	883	173	1.1	50.6
RACCD 0.30	0.30	386.4	126	33.6	-	682	938	184	1.1	59.8
RACCD 0.35	0.35	386.4	147	33.6	-	662	911	179	1.1	55.4
RACCD 0.40	0.40	386.4	168	33.6	-	641	883	173	1.1	50.4

Legend: NC, Normal Concrete; RCA, Recycled Aggregate Concrete; RACCM, Recycled Aggregate Concrete containing Coated RCAs Manually; RACCD, Recycled Aggregate Concrete containing Coated RCAs in Desiccator; C, Cement; W, Water; SF, Silica fume; SP, Superplasticizer.

## Data Availability

Not applicable.
